# Bis[μ_2_-1-(2-carb­oxy­benzo­yl)thio­semi­carbazide(3−)]hexa­pyridine­trinickel(II) pyridine monosolvate monohydrate

**DOI:** 10.1107/S1600536811048367

**Published:** 2011-11-23

**Authors:** Fan Cao, Leilei Li, Dacheng Li

**Affiliations:** a School of Chemistry and Chemical Engineering, Liaocheng University, Shandong 252059, People’s Republic of China

## Abstract

The reaction of Ni(OAc)_2_·4H_2_O with 1-(2-carb­oxy­benzo­yl)thio­semicarbazide (H_3_ *L*) produces the title complex, [Ni_3_(C_9_H_6_N_3_O_3_S)_2_(C_5_H_5_N)_6_]·C_5_H_5_N·2H_2_O, which contains an linear array of three Ni^II^ atoms. The asymmetric unit contains half of the complex mol­ecule, a water mol­ecule and a half-mol­ecule of pyridine. The central Ni^II^ atom, located on a crystallographic inversion centre, has an octa­hedral N_4_O_2_ environment. The other two Ni^II^ atoms have a square-pyramidal N_3_OS environment, each bridged to the central Ni^II^ atom *via* the *L* ^3−^ group. The carboxyl­ate groups coordinate to the metal atoms in a monodentate fashion. The water mol­ecule is linked to the complex mol­ecule *via* O—H⋯O hydrogen bonds. The mol­ecules further assemble into a one-dimensional network parallel to [001] *via* inter­molecular N—H⋯O hydrogen bonds.

## Related literature

For related structures and the synthesis of the 1-(2-carb­oxy­benzo­yl)thio­semicarbazide ligand, see: Shen *et al.* (1997 ▶). 
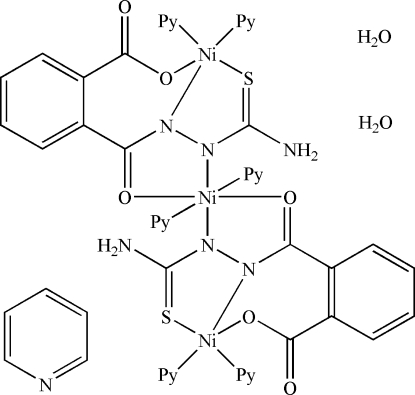


## Experimental

### 

#### Crystal data

[Ni_3_(C_9_H_6_N_3_O_3_S)_2_(C_5_H_5_N)_6_]·C_5_H_5_N·2H_2_O
*M* *_r_* = 1238.32
Monoclinic, 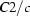

*a* = 34.490 (3) Å
*b* = 8.8510 (7) Å
*c* = 17.8941 (16) Å
β = 90.912 (1)°
*V* = 5461.9 (8) Å^3^
*Z* = 4
Mo *K*α radiation
μ = 1.17 mm^−1^
*T* = 293 K
0.38 × 0.33 × 0.15 mm

#### Data collection

Bruker SMART CCD area-detector diffractometer
Absorption correction: multi-scan (*SADABS*; Sheldrick, 1996 ▶) *T*_min_ = 0.666, *T*_max_ = 0.844
13285 measured reflections
4808 independent reflections
2586 reflections with *I* > 2σ(*I*)
*R*_int_ = 0.073

#### Refinement

*R*[*F* ^2^ > 2σ(*F* ^2^)] = 0.049
*wR*(*F* ^2^) = 0.105
*S* = 0.99
4808 reflections
359 parameters
H-atom parameters constrained
Δρ_max_ = 0.65 e Å^−3^
Δρ_min_ = −0.47 e Å^−3^

### 

Data collection: *SMART* (Bruker, 2007 ▶); cell refinement: *SAINT* (Bruker, 2007 ▶); data reduction: *SAINT*; program(s) used to solve structure: *SHELXS97* (Sheldrick, 2008 ▶); program(s) used to refine structure: *SHELXL97* (Sheldrick, 2008 ▶); molecular graphics: *SHELXTL* (Sheldrick, 2008 ▶); software used to prepare material for publication: *SHELXTL*.

## Supplementary Material

Crystal structure: contains datablock(s) I, global. DOI: 10.1107/S1600536811048367/vm2135sup1.cif

Structure factors: contains datablock(s) I. DOI: 10.1107/S1600536811048367/vm2135Isup2.hkl

Additional supplementary materials:  crystallographic information; 3D view; checkCIF report

## Figures and Tables

**Table 1:** Hydrogen-bond geometry (Å, °)

*D*—H⋯*A*	*D*—H	H⋯*A*	*D*⋯*A*	*D*—H⋯*A*
N3—H3*A*⋯O1^i^	0.86	2.10	2.913 (4)	158
N3—H3*B*⋯O3^ii^	0.86	2.13	2.975 (5)	168
O4—H4*C*⋯O2	0.85	2.41	3.088 (5)	137
O4—H4*D*⋯O3	0.85	2.35	3.020 (5)	136

Symmetry codes: (i) 
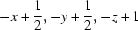
; (ii) 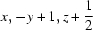.

**Fig. 1.**
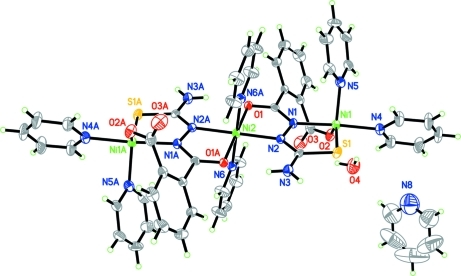
The molecular structure of the title compound, with atom labels and 30% probability displacement ellipsoids (symmetry codes to generate second half of complex and pyridine are -x + 1/2,-y + 1/2,-z + 1 and -x, y, -z + 1/2, respectively).

## supplementary crystallographic information

### Comment

The asymmetric unit of the title complex contains a half complex, a water molecule and a half pyridine molecule (symmetry codes to generate second half of complex and pyridine are -*x* + 1/2,-*y* + 1/2,-*z* + 1 and -*x*, *y*, -*z* + 1/2, respectively). The three nickel atoms are linked together linearly by two µ_2_- bridged L groups. The central Ni2 atom is in a six-coordinated octahedral geometry with two pyridine molecules in axial positions and two amido-carbonyl oxygen atoms and two nitrogen atoms in the equatorial plane. The terminal Ni1 atom is coordinated in a trigonal-bipyramidal geometry composed of two nitrogen atoms from two pyridine molecules, one sulfur atom from the thiourea, one amido-carbonyl nitrogen atom, as well as one oxygen atom from the carboxylate. Thus, the carbonyl oxygen O1 and amine nitrogen N2 atoms of one ligand are bound to Ni2 forming a five-membered chelate ring, while the benzoyloxy oxygen O2, amine nitrogen N1 and sulfydryl sulfur S1 atoms are bound to terminal Ni1 atom forming a five-membered chelate ring and a seven-membered ring. The special position of the central Ni atom generates the linear organization of the three Ni atoms. The molecules further assemble into a one-dimensional network *via* intermolecular N—H···O hydrogen bonds (Table 1).

### Experimental

The title compound, [Ni_3_L_2_(Py)_6_].Py.H_2_O, was synthesized by the reaction of 3 mmol Ni(OAc)_2_.4H_2_O and 2 mmol 1-(2-carboxybenzoyl) thiosemicarbazide (H3L, synthesis described in Shen *et al.*, 1997) in 10 ml methanol and 5 ml pyridine. The solution was stired for 6 hours. After slow evaporation of the solution over one month, deep red crystals suitable for X-ray diffraction were obtained. (yield 42.3%, m.p. 534-538 K).

### Refinement

All H atoms were placed in geometrically idealized positions and treated as riding on their parent atoms, with C—H 0.93, N—H 0.86, and *U*_iso_(H) = 1.2*U*_eq_(C) or *U*_iso_(H) = 1.5*U*_eq_(N). The H atoms of the water molecule were located from the Fourier map and refined constraining the O-H distances at 0.85 Å and *U*_iso_(H) = 1.2*U*_eq_(O) .

### Crystal data

**Table:**

[Ni_3_(C_9_H_6_N_3_O_3_S)_2_(C_5_H_5_N)_6_]·C_5_H_5_N·2H_2_O	*F*(000) = 2560
*M**_r_* = 1238.32	*D*_x_ = 1.506 Mg m^−^^3^
Monoclinic, *C*2/*c*	Mo *K*α radiation, λ = 0.71073 Å
Hall symbol: -C 2yc	Cell parameters from 1906 reflections
*a* = 34.490 (3) Å	θ = 2.3–23.5°
*b* = 8.8510 (7) Å	µ = 1.17 mm^−^^1^
*c* = 17.8941 (16) Å	*T* = 293 K
β = 90.912 (1)°	Block, black
*V* = 5461.9 (8) Å^3^	0.38 × 0.33 × 0.15 mm
*Z* = 4	

### Data collection

**Table:**

Bruker SMART CCD area-detector diffractometer	4808 independent reflections
Radiation source: fine-focus sealed tube	2586 reflections with *I* > 2σ(*I*)
graphite	*R*_int_ = 0.073
phi and ω scans	θ_max_ = 25.0°, θ_min_ = 2.3°
Absorption correction: multi-scan (*SADABS*; Sheldrick, 1996)	*h* = −40→32
*T*_min_ = 0.666, *T*_max_ = 0.844	*k* = −10→10
13285 measured reflections	*l* = −18→21

### Refinement

**Table:**

Refinement on *F*^2^	Primary atom site location: structure-invariant direct methods
Least-squares matrix: full	Secondary atom site location: difference Fourier map
*R*[*F*^2^ > 2σ(*F*^2^)] = 0.049	Hydrogen site location: inferred from neighbouring sites
*wR*(*F*^2^) = 0.105	H-atom parameters constrained
*S* = 0.99	*w* = 1/[σ^2^(*F*_o_^2^) + (0.032*P*)^2^] where *P* = (*F*_o_^2^ + 2*F*_c_^2^)/3
4808 reflections	(Δ/σ)_max_ < 0.001
359 parameters	Δρ_max_ = 0.65 e Å^−^^3^
0 restraints	Δρ_min_ = −0.47 e Å^−^^3^

### Fractional atomic coordinates and isotropic or equivalent isotropic displacement parameters (Å^2^)

**Table:**

	*x*	*y*	*z*	*U*_iso_*/*U*_eq_	
Ni1	0.112323 (15)	0.26236 (7)	0.43526 (3)	0.03118 (19)	
Ni2	0.2500	0.2500	0.5000	0.0262 (2)	
S1	0.11994 (3)	0.37106 (14)	0.55102 (7)	0.0381 (3)	
N1	0.17092 (9)	0.2355 (4)	0.44216 (18)	0.0268 (8)	
N2	0.19160 (9)	0.2959 (4)	0.50367 (19)	0.0267 (9)	
N3	0.18838 (10)	0.4244 (4)	0.61357 (19)	0.0389 (10)	
H3A	0.2132	0.4211	0.6176	0.058*	
H3B	0.1750	0.4680	0.6476	0.058*	
N4	0.05243 (11)	0.3042 (5)	0.4317 (2)	0.0410 (11)	
N5	0.10345 (10)	0.0358 (4)	0.4379 (2)	0.0381 (10)	
N6	0.25781 (10)	0.4472 (4)	0.4275 (2)	0.0323 (9)	
O1	0.22954 (8)	0.1319 (3)	0.41037 (15)	0.0287 (7)	
O2	0.11502 (9)	0.3467 (4)	0.33229 (17)	0.0438 (9)	
O3	0.15374 (11)	0.4067 (4)	0.2398 (2)	0.0701 (12)	
C1	0.17038 (12)	0.3613 (5)	0.5543 (2)	0.0275 (11)	
C2	0.19307 (12)	0.1557 (5)	0.3982 (2)	0.0277 (11)	
C3	0.14056 (14)	0.3153 (6)	0.2833 (3)	0.0432 (13)	
C4	0.17538 (11)	0.0773 (5)	0.3323 (2)	0.0263 (10)	
C5	0.15244 (12)	0.1517 (5)	0.2784 (2)	0.0327 (11)	
C6	0.13875 (14)	0.0710 (7)	0.2166 (3)	0.0513 (14)	
H6	0.1241	0.1200	0.1799	0.062*	
C7	0.14671 (16)	−0.0802 (7)	0.2094 (3)	0.0591 (16)	
H7	0.1369	−0.1337	0.1686	0.071*	
C8	0.16930 (15)	−0.1533 (6)	0.2626 (3)	0.0574 (16)	
H8	0.1746	−0.2559	0.2579	0.069*	
C9	0.18387 (13)	−0.0729 (5)	0.3224 (3)	0.0391 (12)	
H9	0.1999	−0.1215	0.3572	0.047*	
C10	0.02994 (14)	0.2411 (6)	0.4830 (3)	0.0618 (16)	
H10	0.0414	0.1777	0.5184	0.074*	
C11	−0.00927 (16)	0.2651 (8)	0.4862 (4)	0.078 (2)	
H11	−0.0237	0.2186	0.5232	0.094*	
C12	−0.02678 (17)	0.3547 (8)	0.4363 (4)	0.081 (2)	
H12	−0.0534	0.3716	0.4379	0.098*	
C13	−0.00470 (17)	0.4215 (7)	0.3825 (4)	0.076 (2)	
H13	−0.0159	0.4851	0.3469	0.091*	
C14	0.03455 (15)	0.3917 (6)	0.3827 (3)	0.0557 (15)	
H14	0.0493	0.4364	0.3457	0.067*	
C15	0.12039 (13)	−0.0501 (6)	0.4910 (3)	0.0475 (14)	
H15	0.1316	−0.0021	0.5321	0.057*	
C16	0.12199 (16)	−0.2043 (7)	0.4878 (4)	0.0645 (17)	
H16	0.1335	−0.2595	0.5264	0.077*	
C17	0.10612 (17)	−0.2764 (6)	0.4261 (4)	0.0678 (18)	
H17	0.1077	−0.3808	0.4214	0.081*	
C18	0.08817 (15)	−0.1917 (6)	0.3723 (3)	0.0560 (16)	
H18	0.0766	−0.2378	0.3309	0.067*	
C19	0.08739 (13)	−0.0390 (6)	0.3799 (3)	0.0428 (13)	
H19	0.0750	0.0171	0.3427	0.051*	
C20	0.27422 (15)	0.5707 (6)	0.4541 (3)	0.0625 (17)	
H20	0.2822	0.5715	0.5040	0.075*	
C21	0.28014 (16)	0.6987 (6)	0.4121 (4)	0.0709 (19)	
H21	0.2917	0.7836	0.4335	0.085*	
C22	0.26900 (14)	0.6994 (6)	0.3394 (3)	0.0495 (14)	
H22	0.2727	0.7842	0.3096	0.059*	
C23	0.25230 (16)	0.5732 (7)	0.3113 (3)	0.0612 (16)	
H23	0.2443	0.5695	0.2614	0.073*	
C24	0.24728 (15)	0.4514 (6)	0.3566 (3)	0.0562 (15)	
H24	0.2357	0.3658	0.3361	0.067*	
O4	0.07741 (10)	0.5704 (5)	0.2203 (2)	0.0882 (13)	
H4C	0.0751	0.5189	0.2601	0.106*	
H4D	0.0999	0.5533	0.2036	0.106*	
N8	0.0000	0.8034 (18)	0.2500	0.172 (5)	
C25	0.0223 (3)	0.8744 (16)	0.2004 (7)	0.159 (5)	
H25	0.0367	0.8213	0.1657	0.191*	
C26	0.0227 (5)	1.0234 (17)	0.2033 (11)	0.222 (10)	
H26	0.0394	1.0749	0.1719	0.266*	
C27	0.0000	1.106 (3)	0.2500	0.28 (3)	
H27	0.0000	1.2110	0.2500	0.339*	

### Atomic displacement parameters (Å^2^)

**Table:**

	*U*^11^	*U*^22^	*U*^33^	*U*^12^	*U*^13^	*U*^23^
Ni1	0.0281 (3)	0.0351 (4)	0.0305 (4)	0.0010 (3)	0.0041 (3)	0.0008 (3)
Ni2	0.0239 (4)	0.0297 (5)	0.0251 (5)	−0.0005 (4)	0.0025 (3)	−0.0042 (4)
S1	0.0323 (7)	0.0486 (8)	0.0336 (7)	0.0086 (6)	0.0070 (5)	−0.0043 (6)
N1	0.024 (2)	0.037 (2)	0.0192 (19)	0.0002 (18)	0.0037 (15)	−0.0073 (18)
N2	0.027 (2)	0.030 (2)	0.023 (2)	−0.0005 (16)	0.0031 (17)	−0.0047 (18)
N3	0.034 (2)	0.053 (3)	0.030 (2)	0.001 (2)	0.0039 (18)	−0.016 (2)
N4	0.032 (2)	0.050 (3)	0.041 (3)	−0.002 (2)	0.004 (2)	0.000 (2)
N5	0.036 (2)	0.045 (3)	0.034 (3)	−0.006 (2)	0.0023 (19)	0.001 (2)
N6	0.031 (2)	0.033 (2)	0.033 (2)	0.0000 (18)	0.0058 (18)	0.002 (2)
O1	0.0252 (18)	0.0301 (18)	0.0309 (18)	0.0012 (14)	0.0002 (13)	−0.0051 (15)
O2	0.041 (2)	0.056 (2)	0.034 (2)	0.0098 (17)	0.0117 (16)	0.0110 (18)
O3	0.083 (3)	0.064 (3)	0.065 (3)	0.010 (2)	0.036 (2)	0.034 (2)
C1	0.032 (3)	0.026 (3)	0.025 (3)	0.006 (2)	0.003 (2)	−0.005 (2)
C2	0.029 (3)	0.023 (3)	0.031 (3)	−0.006 (2)	0.002 (2)	0.004 (2)
C3	0.035 (3)	0.058 (4)	0.036 (3)	−0.001 (3)	−0.004 (3)	0.009 (3)
C4	0.024 (3)	0.034 (3)	0.021 (3)	−0.004 (2)	0.0070 (19)	−0.009 (2)
C5	0.029 (3)	0.046 (3)	0.023 (3)	−0.006 (2)	0.007 (2)	−0.002 (2)
C6	0.051 (4)	0.072 (4)	0.031 (3)	−0.010 (3)	−0.003 (2)	0.000 (3)
C7	0.068 (4)	0.074 (5)	0.035 (4)	−0.017 (3)	0.000 (3)	−0.029 (3)
C8	0.061 (4)	0.050 (4)	0.062 (4)	0.002 (3)	−0.001 (3)	−0.029 (3)
C9	0.039 (3)	0.039 (3)	0.039 (3)	0.003 (2)	0.005 (2)	−0.015 (3)
C10	0.036 (3)	0.082 (5)	0.068 (4)	0.007 (3)	0.007 (3)	0.016 (4)
C11	0.036 (4)	0.108 (6)	0.092 (5)	0.007 (4)	0.020 (3)	0.017 (4)
C12	0.030 (4)	0.100 (6)	0.115 (6)	0.014 (4)	0.012 (4)	0.000 (5)
C13	0.044 (4)	0.092 (5)	0.093 (5)	0.021 (4)	−0.009 (4)	0.014 (4)
C14	0.041 (3)	0.062 (4)	0.065 (4)	0.003 (3)	0.002 (3)	0.010 (3)
C15	0.051 (4)	0.053 (4)	0.039 (3)	−0.007 (3)	0.000 (3)	0.004 (3)
C16	0.070 (4)	0.045 (4)	0.078 (5)	0.001 (3)	0.000 (3)	0.022 (3)
C17	0.078 (5)	0.027 (3)	0.099 (5)	−0.007 (3)	0.019 (4)	0.001 (4)
C18	0.051 (4)	0.048 (4)	0.069 (4)	−0.018 (3)	0.007 (3)	−0.014 (3)
C19	0.035 (3)	0.046 (4)	0.047 (4)	−0.009 (2)	0.005 (2)	−0.004 (3)
C20	0.080 (4)	0.048 (4)	0.059 (4)	−0.016 (3)	−0.018 (3)	0.005 (3)
C21	0.085 (5)	0.043 (4)	0.084 (5)	−0.025 (3)	−0.019 (4)	0.014 (4)
C22	0.053 (4)	0.043 (4)	0.053 (4)	0.002 (3)	0.013 (3)	0.018 (3)
C23	0.076 (4)	0.066 (4)	0.042 (4)	−0.009 (3)	0.001 (3)	0.006 (3)
C24	0.083 (4)	0.051 (4)	0.035 (3)	−0.021 (3)	0.003 (3)	0.004 (3)
O4	0.077 (3)	0.111 (4)	0.076 (3)	0.015 (3)	0.011 (2)	0.011 (3)
N8	0.155 (14)	0.194 (17)	0.164 (15)	0.000	−0.023 (10)	0.000
C25	0.157 (12)	0.174 (12)	0.146 (11)	−0.012 (11)	−0.020 (8)	0.040 (11)
C26	0.253 (18)	0.17 (2)	0.23 (2)	−0.107 (18)	−0.143 (14)	0.094 (17)
C27	0.46 (6)	0.10 (2)	0.28 (5)	0.000	−0.29 (4)	0.000

### Geometric parameters (Å, °)

**Table:**

Ni1—O2	1.992 (3)	C9—H9	0.9300
Ni1—N5	2.029 (4)	C10—C11	1.371 (6)
Ni1—N1	2.037 (3)	C10—H10	0.9300
Ni1—N4	2.099 (4)	C11—C12	1.332 (8)
Ni1—S1	2.2953 (13)	C11—H11	0.9300
Ni2—O1	2.032 (3)	C12—C13	1.371 (8)
Ni2—O1^i^	2.032 (3)	C12—H12	0.9300
Ni2—N2^i^	2.057 (3)	C13—C14	1.379 (6)
Ni2—N2	2.057 (3)	C13—H13	0.9300
Ni2—N6	2.194 (4)	C14—H14	0.9300
Ni2—N6^i^	2.194 (4)	C15—C16	1.367 (7)
S1—C1	1.742 (4)	C15—H15	0.9300
N1—C2	1.311 (5)	C16—C17	1.380 (7)
N1—N2	1.408 (4)	C16—H16	0.9300
N2—C1	1.309 (5)	C17—C18	1.361 (7)
N3—C1	1.342 (5)	C17—H17	0.9300
N3—H3A	0.8600	C18—C19	1.359 (6)
N3—H3B	0.8600	C18—H18	0.9300
N4—C14	1.317 (6)	C19—H19	0.9300
N4—C10	1.334 (6)	C20—C21	1.377 (7)
N5—C19	1.343 (5)	C20—H20	0.9300
N5—C15	1.343 (5)	C21—C22	1.351 (7)
N6—C24	1.314 (5)	C21—H21	0.9300
N6—C20	1.317 (6)	C22—C23	1.350 (6)
O1—C2	1.290 (4)	C22—H22	0.9300
O2—C3	1.283 (5)	C23—C24	1.362 (7)
O3—C3	1.216 (5)	C23—H23	0.9300
C2—C4	1.492 (5)	C24—H24	0.9300
C3—C5	1.508 (6)	O4—H4C	0.8501
C4—C9	1.374 (6)	O4—H4D	0.8498
C4—C5	1.402 (6)	N8—C25^ii^	1.338 (11)
C5—C6	1.392 (6)	N8—C25	1.338 (11)
C6—C7	1.373 (7)	C25—C26	1.320 (16)
C6—H6	0.9300	C25—H25	0.9300
C7—C8	1.381 (7)	C26—C27	1.366 (19)
C7—H7	0.9300	C26—H26	0.9300
C8—C9	1.374 (6)	C27—C26^ii^	1.366 (19)
C8—H8	0.9300	C27—H27	0.9300
			
O2—Ni1—N5	113.67 (15)	C6—C7—H7	119.9
O2—Ni1—N1	92.23 (13)	C8—C7—H7	119.9
N5—Ni1—N1	91.89 (14)	C9—C8—C7	119.3 (5)
O2—Ni1—N4	88.08 (14)	C9—C8—H8	120.3
N5—Ni1—N4	91.52 (15)	C7—C8—H8	120.3
N1—Ni1—N4	176.13 (14)	C4—C9—C8	121.6 (5)
O2—Ni1—S1	132.18 (10)	C4—C9—H9	119.2
N5—Ni1—S1	114.10 (12)	C8—C9—H9	119.2
N1—Ni1—S1	83.98 (10)	N4—C10—C11	123.3 (5)
N4—Ni1—S1	92.95 (11)	N4—C10—H10	118.4
O1—Ni2—O1^i^	180.000 (1)	C11—C10—H10	118.4
O1—Ni2—N2^i^	101.60 (12)	C12—C11—C10	120.1 (6)
O1^i^—Ni2—N2^i^	78.40 (12)	C12—C11—H11	120.0
O1—Ni2—N2	78.40 (12)	C10—C11—H11	120.0
O1^i^—Ni2—N2	101.60 (12)	C11—C12—C13	118.5 (6)
N2^i^—Ni2—N2	180.000 (1)	C11—C12—H12	120.8
O1—Ni2—N6	89.26 (13)	C13—C12—H12	120.8
O1^i^—Ni2—N6	90.74 (13)	C12—C13—C14	118.2 (6)
N2^i^—Ni2—N6	90.51 (13)	C12—C13—H13	120.9
N2—Ni2—N6	89.49 (13)	C14—C13—H13	120.9
O1—Ni2—N6^i^	90.74 (13)	N4—C14—C13	124.3 (5)
O1^i^—Ni2—N6^i^	89.25 (13)	N4—C14—H14	117.9
N2^i^—Ni2—N6^i^	89.49 (13)	C13—C14—H14	117.9
N2—Ni2—N6^i^	90.51 (13)	N5—C15—C16	123.6 (5)
N6—Ni2—N6^i^	180.000 (1)	N5—C15—H15	118.2
C1—S1—Ni1	96.29 (15)	C16—C15—H15	118.2
C2—N1—N2	112.4 (3)	C15—C16—C17	118.6 (5)
C2—N1—Ni1	127.9 (3)	C15—C16—H16	120.7
N2—N1—Ni1	119.5 (2)	C17—C16—H16	120.7
C1—N2—N1	115.3 (3)	C18—C17—C16	118.8 (5)
C1—N2—Ni2	131.9 (3)	C18—C17—H17	120.6
N1—N2—Ni2	112.6 (2)	C16—C17—H17	120.6
C1—N3—H3A	120.0	C19—C18—C17	119.1 (5)
C1—N3—H3B	120.0	C19—C18—H18	120.5
H3A—N3—H3B	120.0	C17—C18—H18	120.5
C14—N4—C10	115.7 (4)	N5—C19—C18	124.0 (5)
C14—N4—Ni1	125.1 (4)	N5—C19—H19	118.0
C10—N4—Ni1	119.2 (4)	C18—C19—H19	118.0
C19—N5—C15	115.9 (4)	N6—C20—C21	123.5 (5)
C19—N5—Ni1	122.0 (3)	N6—C20—H20	118.3
C15—N5—Ni1	120.8 (3)	C21—C20—H20	118.3
C24—N6—C20	115.9 (4)	C22—C21—C20	119.2 (5)
C24—N6—Ni2	124.0 (3)	C22—C21—H21	120.4
C20—N6—Ni2	120.2 (3)	C20—C21—H21	120.4
C2—O1—Ni2	112.0 (3)	C23—C22—C21	117.9 (5)
C3—O2—Ni1	126.4 (3)	C23—C22—H22	121.0
N2—C1—N3	118.3 (4)	C21—C22—H22	121.0
N2—C1—S1	124.6 (3)	C22—C23—C24	119.4 (5)
N3—C1—S1	117.1 (3)	C22—C23—H23	120.3
O1—C2—N1	124.3 (4)	C24—C23—H23	120.3
O1—C2—C4	116.3 (4)	N6—C24—C23	124.2 (5)
N1—C2—C4	119.3 (4)	N6—C24—H24	117.9
O3—C3—O2	124.1 (5)	C23—C24—H24	117.9
O3—C3—C5	119.8 (5)	H4C—O4—H4D	107.3
O2—C3—C5	116.0 (4)	C25^ii^—N8—C25	124 (2)
C9—C4—C5	119.1 (4)	C26—C25—N8	116.7 (19)
C9—C4—C2	117.8 (4)	C26—C25—H25	121.6
C5—C4—C2	123.1 (4)	N8—C25—H25	121.6
C6—C5—C4	119.1 (5)	C25—C26—C27	123 (3)
C6—C5—C3	116.8 (4)	C25—C26—H26	118.3
C4—C5—C3	124.1 (4)	C27—C26—H26	118.3
C7—C6—C5	120.6 (5)	C26^ii^—C27—C26	115 (3)
C7—C6—H6	119.7	C26^ii^—C27—H27	122.3
C5—C6—H6	119.7	C26—C27—H27	122.3
C6—C7—C8	120.2 (5)		
			
O2—Ni1—S1—C1	−83.36 (19)	N1—N2—C1—N3	−178.5 (3)
N5—Ni1—S1—C1	93.57 (18)	Ni2—N2—C1—N3	6.7 (6)
N1—Ni1—S1—C1	4.19 (18)	N1—N2—C1—S1	2.2 (5)
N4—Ni1—S1—C1	−173.44 (18)	Ni2—N2—C1—S1	−172.6 (2)
O2—Ni1—N1—C2	−58.4 (4)	Ni1—S1—C1—N2	−4.9 (4)
N5—Ni1—N1—C2	55.3 (4)	Ni1—S1—C1—N3	175.8 (3)
N4—Ni1—N1—C2	−153 (2)	Ni2—O1—C2—N1	−5.8 (5)
S1—Ni1—N1—C2	169.4 (4)	Ni2—O1—C2—C4	178.4 (3)
O2—Ni1—N1—N2	127.7 (3)	N2—N1—C2—O1	1.4 (6)
N5—Ni1—N1—N2	−118.6 (3)	Ni1—N1—C2—O1	−172.8 (3)
N4—Ni1—N1—N2	33 (2)	N2—N1—C2—C4	177.1 (3)
S1—Ni1—N1—N2	−4.5 (3)	Ni1—N1—C2—C4	2.8 (6)
C2—N1—N2—C1	−172.1 (4)	Ni1—O2—C3—O3	−143.0 (4)
Ni1—N1—N2—C1	2.7 (4)	Ni1—O2—C3—C5	40.6 (6)
C2—N1—N2—Ni2	3.7 (4)	O1—C2—C4—C9	45.9 (5)
Ni1—N1—N2—Ni2	178.48 (16)	N1—C2—C4—C9	−130.1 (4)
O1—Ni2—N2—C1	169.9 (4)	O1—C2—C4—C5	−130.6 (4)
O1^i^—Ni2—N2—C1	−10.1 (4)	N1—C2—C4—C5	53.4 (6)
N2^i^—Ni2—N2—C1	34 (81)	C9—C4—C5—C6	0.3 (6)
N6—Ni2—N2—C1	−100.7 (4)	C2—C4—C5—C6	176.7 (4)
N6^i^—Ni2—N2—C1	79.3 (4)	C9—C4—C5—C3	178.9 (4)
O1—Ni2—N2—N1	−5.0 (2)	C2—C4—C5—C3	−4.7 (7)
O1^i^—Ni2—N2—N1	175.0 (2)	O3—C3—C5—C6	−70.0 (6)
N2^i^—Ni2—N2—N1	−141 (81)	O2—C3—C5—C6	106.5 (5)
N6—Ni2—N2—N1	84.3 (3)	O3—C3—C5—C4	111.4 (6)
N6^i^—Ni2—N2—N1	−95.7 (3)	O2—C3—C5—C4	−72.1 (6)
O2—Ni1—N4—C14	−15.1 (4)	C4—C5—C6—C7	1.7 (7)
N5—Ni1—N4—C14	−128.8 (4)	C3—C5—C6—C7	−177.0 (5)
N1—Ni1—N4—C14	80 (2)	C5—C6—C7—C8	−1.7 (8)
S1—Ni1—N4—C14	117.0 (4)	C6—C7—C8—C9	−0.3 (8)
O2—Ni1—N4—C10	166.0 (4)	C5—C4—C9—C8	−2.4 (7)
N5—Ni1—N4—C10	52.4 (4)	C2—C4—C9—C8	−179.0 (4)
N1—Ni1—N4—C10	−99 (2)	C7—C8—C9—C4	2.4 (8)
S1—Ni1—N4—C10	−61.9 (4)	C14—N4—C10—C11	−0.6 (8)
O2—Ni1—N5—C19	−22.5 (4)	Ni1—N4—C10—C11	178.3 (4)
N1—Ni1—N5—C19	−115.8 (3)	N4—C10—C11—C12	0.3 (10)
N4—Ni1—N5—C19	66.1 (4)	C10—C11—C12—C13	−0.1 (10)
S1—Ni1—N5—C19	160.0 (3)	C11—C12—C13—C14	0.3 (10)
O2—Ni1—N5—C15	143.8 (3)	C10—N4—C14—C13	0.9 (8)
N1—Ni1—N5—C15	50.5 (4)	Ni1—N4—C14—C13	−178.0 (4)
N4—Ni1—N5—C15	−127.7 (4)	C12—C13—C14—N4	−0.8 (9)
S1—Ni1—N5—C15	−33.7 (4)	C19—N5—C15—C16	0.5 (7)
O1—Ni2—N6—C24	4.2 (4)	Ni1—N5—C15—C16	−166.6 (4)
O1^i^—Ni2—N6—C24	−175.8 (4)	N5—C15—C16—C17	1.4 (8)
N2^i^—Ni2—N6—C24	105.8 (4)	C15—C16—C17—C18	−2.5 (8)
N2—Ni2—N6—C24	−74.2 (4)	C16—C17—C18—C19	1.8 (8)
N6^i^—Ni2—N6—C24	168 (100)	C15—N5—C19—C18	−1.2 (7)
O1—Ni2—N6—C20	−175.2 (4)	Ni1—N5—C19—C18	165.7 (4)
O1^i^—Ni2—N6—C20	4.8 (4)	C17—C18—C19—N5	0.1 (8)
N2^i^—Ni2—N6—C20	−73.6 (4)	C24—N6—C20—C21	0.5 (8)
N2—Ni2—N6—C20	106.4 (4)	Ni2—N6—C20—C21	179.9 (4)
N6^i^—Ni2—N6—C20	−11 (100)	N6—C20—C21—C22	−0.5 (9)
O1^i^—Ni2—O1—C2	132 (100)	C20—C21—C22—C23	0.2 (8)
N2^i^—Ni2—O1—C2	−174.4 (3)	C21—C22—C23—C24	0.1 (8)
N2—Ni2—O1—C2	5.6 (3)	C20—N6—C24—C23	−0.2 (8)
N6—Ni2—O1—C2	−84.0 (3)	Ni2—N6—C24—C23	−179.5 (4)
N6^i^—Ni2—O1—C2	96.0 (3)	C22—C23—C24—N6	−0.2 (9)
N5—Ni1—O2—C3	−63.3 (4)	C25^ii^—N8—C25—C26	2.2 (9)
N1—Ni1—O2—C3	29.7 (4)	N8—C25—C26—C27	−4.6 (18)
N4—Ni1—O2—C3	−154.1 (4)	C25—C26—C27—C26^ii^	2.4 (10)
S1—Ni1—O2—C3	113.6 (4)		

Symmetry codes: (i) −*x*+1/2, −*y*+1/2, −*z*+1; (ii) −*x*, *y*, −*z*+1/2.

### Hydrogen-bond geometry (Å, °)

**Table:**

*D*—H···*A*	*D*—H	H···*A*	*D*···*A*	*D*—H···*A*
N3—H3A···O1^i^	0.86	2.10	2.913 (4)	158
N3—H3B···O3^iii^	0.86	2.13	2.975 (5)	168
O4—H4C···O2	0.85	2.41	3.088 (5)	137
O4—H4D···O3	0.85	2.35	3.020 (5)	136

Symmetry codes: (i) −*x*+1/2, −*y*+1/2, −*z*+1; (iii) *x*, −*y*+1, *z*+1/2.

## References

[bb1] Bruker (2007). *SMART* and *SAINT* Bruker AXS Inc., Madison, Wisconsin, USA.

[bb2] Sheldrick, G. M. (1996). *SADABS* University of Göttingen, Germany.

[bb3] Sheldrick, G. M. (2008). *Acta Cryst.* A**64**, 112–122. 10.1107/S0108767307043930 18156677

[bb4] Shen, X., Wu, D. & Huang, X. (1997). *Polyhedron*, **16**, 1477–1482.

